# Effects of the intensified frequency and time ranges on consonant enhancement in bilateral cochlear implant and hearing aid users

**DOI:** 10.3389/fpsyg.2022.918914

**Published:** 2022-08-16

**Authors:** Yang-Soo Yoon, Carrie Drew

**Affiliations:** Laboratory of Translational Auditory Research, Department of Communication Sciences and Disorders, Baylor University, Waco, TX, United States

**Keywords:** consonant recognition, spectral cues, temporal cues, articulation-index gram, binaural integration

## Abstract

A previous study demonstrated that consonant recognition improved significantly in normal hearing listeners when useful frequency and time ranges were intensified by 6 dB. The goal of this study was to determine whether bilateral cochlear implant (BCI) and bilateral hearing aid (BHA) users experienced similar enhancement on consonant recognition with these intensified spectral and temporal cues in noise. In total, 10 BCI and 10 BHA users participated in a recognition test using 14 consonants. For each consonant, we used the frequency and time ranges that are critical for its recognition (called “target frequency and time range”), identified from normal hearing listeners. Then, a signal processing tool called the articulation-index gram (AI-Gram) was utilized to add a 6 dB gain to target frequency and time ranges. Consonant recognition was monaurally and binaurally measured under two signal processing conditions, unprocessed and intensified target frequency and time ranges at +5 and +10 dB signal-to-noise ratio and in quiet conditions. We focused on three comparisons between the BCI and BHA groups: (1) AI-Gram benefits (i.e., before and after intensifying target ranges by 6 dB), (2) enhancement in binaural benefits (better performance with bilateral devices compared to the better ear alone) via the AI-Gram processing, and (3) reduction in binaural interferences (poorer performance with bilateral devices compared to the better ear alone) via the AI-Gram processing. The results showed that the mean AI-Gram benefit was significantly improved for the BCI (max 5.9%) and BHA (max 5.2%) groups. However, the mean binaural benefit was not improved after AI-Gram processing. Individual data showed wide ranges of the AI-Gram benefit (max −1 to 23%) and binaural benefit (max −7.6 to 13%) for both groups. Individual data also showed a decrease in binaural interference in both groups after AI-Gram processing. These results suggest that the frequency and time ranges, intensified by the AI-Gram processing, contribute to consonant enhancement for monaural and binaural listening and both BCI and BHA technologies. The intensified frequency and time ranges helped to reduce binaural interference but contributed less to the synergistic binaural benefit in consonant recognition for both groups.

## Introduction

Recently, our laboratory conducted a study with normal hearing (NH) listeners to assess the effect of specific frequency and time ranges on consonant recognition (Yoon, 2021). In that study, consonant confusion matrices were measured for each of the 14 consonant phonemes in a context consonant + /a/ in quiet. Specific frequency and time ranges contributing to consonant enhancement (which we shall call “target frequency range” and “target time ranges,” respectively.) were identified. Other specific frequency and time ranges of consonants causing consonant confusions (we shall call these “conflicting frequency ranges” and “conflicting time ranges,” respectively) were also identified. Using the articulation-index gram (AI-Gram), a signal processing tool, the target ranges were intensified with a 6 dB gain while the conflicting ranges were removed. Then, consonant recognition was binaurally measured in noise under three signal processing conditions: unprocessed, intensified target ranges, and combined intensified target while removing conflicting ranges. The results showed that consonant recognition improved significantly with intensified target ranges but greatly deteriorated with the combined target-conflicting condition. These findings led to our prediction that improved consonant recognition can be achieved if the hearing device users can detect and integrate these spectral and temporal ranges intensified by the AI-Gram approach. The objective of this study was to apply the intensified target ranges, via the AI-gram on bilateral cochlear implant (BCI) and bilateral hearing aid (BHA) users and determine whether they experienced the similar enhancement on consonant recognition when the target frequency and time ranges were intensified by the AI-Gram. We did not apply the conflicting ranges here because it introduced detrimental effects on consonant recognition in the NH study (Yoon, 2021).

The BCI and BHA groups were utilized to determine whether binaural benefit (better performance with bilateral devices compared to the better ear alone) in speech perception was improved and if binaural interference (poorer performance with bilateral devices compared to the better ear alone) was lessened via the AI-Gram processing. A majority of BCI and BHA users receive binaural benefits in speech perception when compared to monaural users (Ching et al., 2006; Litovsky et al., 2006; Schilder et al., 2017). However, there are significant variabilities within these users when it comes to binaural benefit in speech perception. One potential reason for variability in binaural benefit is the differing abilities of users to process the frequency and time acoustic information that are critical for speech perception (Obuchi et al., 2015; Goupell et al., 2018). Both BCI and BHA users must detect and integrate frequency and time cues that are processed independently by each cochlear implant (CI) or each hearing aid (HA). Although there are different variables that might limit spectral integration between BCI and BHA users, this study focused on determining the effect of intensified target frequency and time ranges on the ability of BCI and BHA listeners to detect and integrate important acoustic cues, resulting in the improved consonant recognition.

Since there are differences in the stimulation of the auditory nerve and the central auditory system processing between BCI and BHA groups, mechanisms for integrating target frequency and time acoustics for consonant recognition may be different between the two groups. Due to the different degrees of reduction in the spiral ganglion neurons, shrinkage of the perikaryon of neurons, and reduced spontaneous activity, the organization of input signal into the auditory cortex is different between electric and acoustic stimulation (Calford, 2002; Irvine and Wright, 2005). Physiological evidence suggests that these differences affect integration in the superior olivary complex or higher nuclei in the central auditory pathway (McPherson and Starr, 1993; Happel et al., 2010). The different effects of long-term electric and acoustic stimulation on peripheral and central auditory processing are also an important factor for influencing the ability to integrate auditory information (Gstoettner et al., 2006; Kronenberger et al., 2014; Skarzynski, 2014). Intense, long-term electric stimulation could cause damage in the outer hair cells and the efferent functionality of the cochlear nerve, particularly the apical regions, which results in negative neural processing in the higher auditory system (Dodson et al., 1986). One specific factor that affects integration for BCI users is binaural spectral mismatch, which can be evoked by various insertion depths of the electrode array into the cochlea of each ear (Yoon et al., 2011; Mukherjee et al., 2012; Mertens et al., 2020). Yoon et al. revealed that speech information was best integrated when the interaural difference in insertion depth of the electrode array was 1 mm or less. This result was seen in both quiet and noisy environments. For the BHA users, asymmetric hearing loss, which is defined as an interaural asymmetry of ≥20 dB HL at two contiguous frequencies over the frequency range of 0.25–8.0 kHz, creates a listening environment of spectral integration across ears (Algom et al., 1989; Ronan et al., 2004; Hall et al., 2008; Yang and Zeng, 2013; Bonnard et al., 2018). These differences between the two groups can result in different listening strategies for spectral and temporal integration.

As an opposing concept to binaural integration, binaural interference can occur with binaural listening. This interference may lead to poorer speech perception. Goupell et al. showed that nine of their BCI adult subjects experienced interference in speech perception both in noise and in quiet (Goupell et al., 2018). In total, five subjects experienced asymmetric contralateral interference, whereas four subjects experienced symmetric contralateral interference. A case study of a preschool aged child who initially utilized monaural amplification and then later utilized a binaural fitting provides an illustration of binaural interference (Schoepflin, 2007). Word recognition scores in the ear that was initially aided were significantly better than those in the unaided ear. This significant difference in word recognition ability between ears was hypothesized to be a result of the effects of auditory deprivation to the unaided ear. After the child was fitted with a second HA, the patient's monaural aided word recognition score was 90% with the ear that was originally aided, compared to a score of 36% in a monaural aided condition with the second ear to be aided. A binaural aided condition yielded a word recognition score of 56%. This indicates that amplification in the poorer ear interfered with word recognition from the better ear, which resulted in poorer binaural performance. The results from these studies suggest that binaural hearing can be negatively impacted by binaural interference, which may be one of the major reasons for variability in binaural benefit.

Determining the potential for binaural benefit and binaural interference is important for optimizing CI and HA outcomes. In this study, we used the AI-Gram processed target frequency and time ranges critical for consonant recognition and determined if BCI and BHA groups displayed improved consonant recognition. Specifically, we designed the study to answer the following three questions: (1) Was there a significant AI-Gram benefit (performance difference in consonant recognition before and after the 6 dB was added to target frequency and time ranges)? (2) Was there a binaural benefit improvement with AI-Gram processing? (3) Was binaural interference reduced with AI-Gram processing?

## Methods

### Subjects

**Figure 1:**
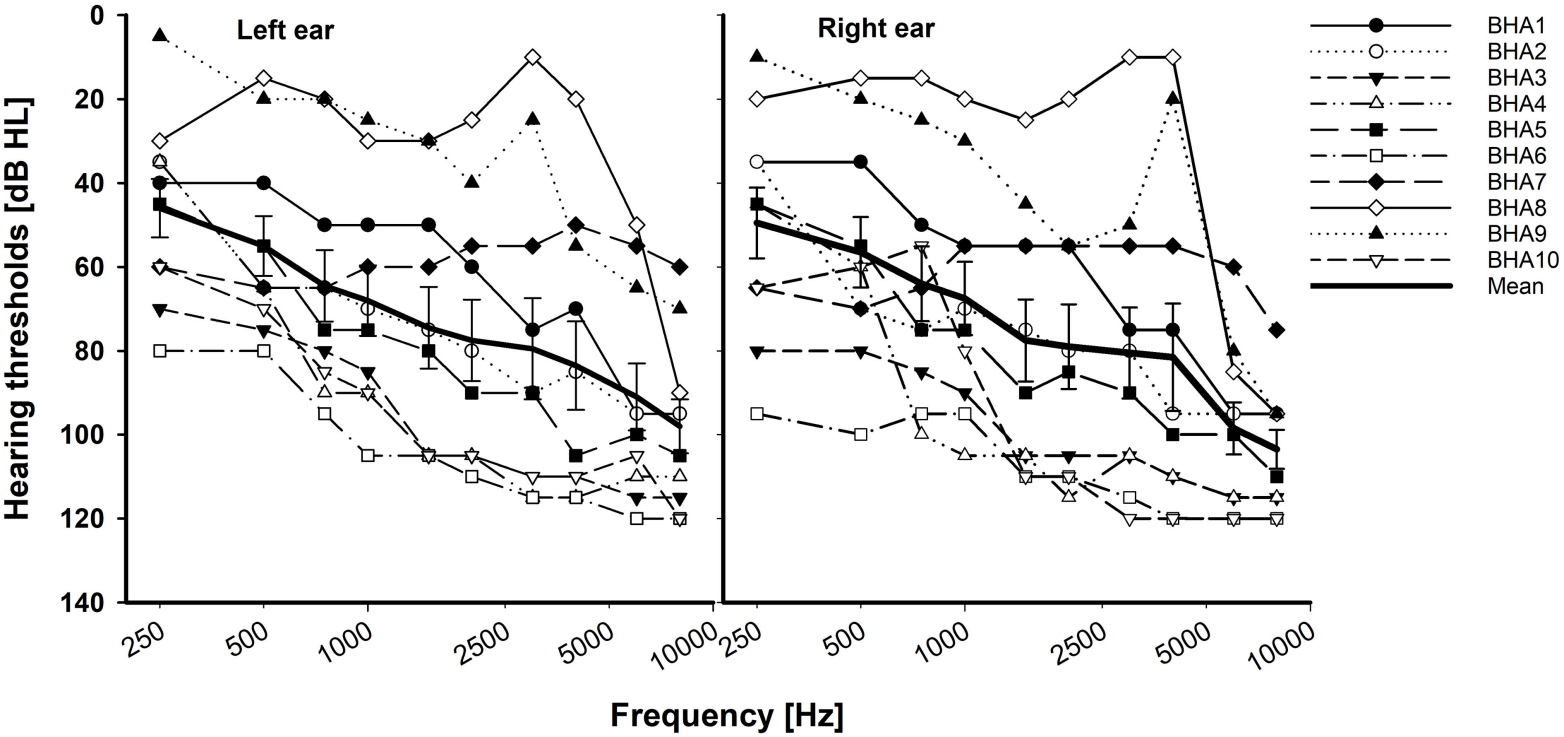
Individual and mean unaided hearing thresholds with standard error for the BHA group. X-axis is a logarithmic scale.

In total, 10 adult BCI users (7 women and 3 men; age range, 19–64 years, mean = 48 years) and 10 BHA adult users (6 women and 4 men; age range, 21–66 years, mean = 39 years) participated in this study. All participants were the native American English speakers and post-lingually deafened. The subjects in both groups had at least 1 year of prior binaural hearing experience. Based on the criteria defining asymmetric hearing loss as the interaural asymmetry of ≥20 dB hearing level at two contiguous frequencies or ≥15 dB hearing level at any two frequencies between 2 and 8 kHz (Durakovic et al., 2019), our BHA group had symmetrical hearing loss. Their unaided hearing thresholds are given in Figure 1. Based on the standard audiogram classification (Bisgaard et al., 2010), all ten BHA subjects were in the sloping hearing loss group: BHA8 and BHA9 were mild, BHA1 was moderate, BHA7 was moderate/severe, BHA2, BHA3, and BHA5 were severe, and BHA4, BHA6, and BHA10 were profound. No information was available regarding the insertion depth of the electrode array for any of the BCI subjects. Data were collected regarding the years of HA or CI experience which provided information about the length of time the peripheral and central auditory pathways were exposed to acoustic or electric stimulation. Information regarding the age of onset of hearing loss was not requested, so length of auditory deprivation between diagnosis and intervention was not available. We did not measure aided hearing thresholds because the BHA subjects were tested using their devices, which were fitted and then verified by matching or closely approximating NAL-NL2 or NAL-NL1 targets using real ear measures by their audiologist. The BCI users' mapping was determined to be appropriate by their audiologist using validated tools such as speech perception testing, sound-field thresholds, various questionnaires, and/or an objective measure (cortical evoked potentials). The BCI and BHA subject's demographic data are given in Tables 1, 2, respectively. All subjects provided written informed consent, and all research protocols were approved by the Texas Tech University Health Sciences Center Institutional Review Board (IRB #L14-048).

**Table 1:** Demographics of the BCI group.

**ID**	**Gender; age at testing**	**Sound Processor; ICS**	**Years of use**	**Presentation level in dB(A) SPL**	**Etiology**
BCI1	F; 32	R: Nucleus 5; Nucleus CI500	1	55	Unknown
		L: Nucleus 5; Nucleus CI500	1		
BCI2	M; 19	R: Nucleus 6; Nucleus CI512	9	55	Unknown
		L: Nucleus 6; Nucleus CI512	9		
BCI3	F; 59	R: Nucleus 6; Nucleus CI512	12	55	Unknown
		L: Nucleus 6; Nucleus CI512	12		
BCI4	F; 50	R: Naida; HiRes Ultra	7	55	German measles
		L: Naida; HiRes Ultra	7		
BCI5	F; 64	R: Nucleus Freedom; Nucleus CI24M	10	65	Otosclerosis
		L: Nucleus Freedom; Nucleus CI24M	9		
BCI6	F; 49	R: Nucleus Freedom; Nucleus CI24M	9	55	Meningitis
		L: Nucleus Freedom; Nucleus CI24M	8		
BCI7	F; 54	R: Harmony; HiRes 90k	6	60	Hereditary
		L: Harmony; HiRes 90k	4		
BCI8	F; 59	R: Nucleus Freedom; Nucleus CI24M	7	65	Unknown
		L: Nucleus Freedom; Nucleus CI24M	5		
BCI9	M; 37	R: Harmony; HiRes 90k	10	65	Ototoxicity
		L: Harmony; HiRes 90k	8		
BCI10	M; 57	R: Nucleus Freedom; Nucleus CI24M	9	55	Unknown
		L: Nucleus Freedom; Nucleus CI24M	9		

*R and L indicate right ear and left ear, respectively. ICS stands for implantable cochlear stimulator*.

**Table 2:** Demographics of the BHA group.

**ID**	**Gender; age at testing**	**Device**	**Years of use**	**Presentation level in dB(A) SPL**	**Etiology**
BHA1	F; 22	R: Phonak Savia	12	70	Waardenburg
		L: Phonak Savia	12		
BHA2	M; 21	R: Phonak 313	15	75	Rubella
		L: Phonak 313	15		
BHA3	F; 34	R: Unitron Moxi Kiss	9	65	Unknown
		L: Unitron Moxi Kiss	12		
BHA4	M; 43	R: Phonak Bolero B	19	65	Unknown
		L: Phonak Bolero B	19		
BHA5	F; 49	R: Oticon Dual Xw Rite	21	70	Unknown
		L: Oticon Dual Wx Rite	21		
BHA6	M; 43	R: Widex Senso	15	L: 85; R & Both: 75	Noise-induced
		L: Widex Senso	19		
BHA7	F; 32	R: Unitron Quantum1	3	70	Unknown
		L: Unitron Quantum1	3		
BHA8	F; 22	R: Phonak Naida Q90	20	L: 65; R & both: 75	Unknown
		L: Phonak Naida Q90	20		
BHA9	M; 66	R: Resound Metrix	13	65	Hereditary
		L: Resound Metrix	13		
BHA10	F; 57	R: Phonak, Supero	7	65	Unknown
		L: Phonak, Supero	10		

*R and L indicate right ear and left ear, respectively*.

### Stimuli

Closed set testing was administered for both subject groups with fourteen of the most frequently used consonants in American English (Hayden, 1950). The consonants were produced by a female talker (fundamental frequency: 228 Hz). The common vowel /a/ was used to produce the stimuli: /pa, ba, ta, da, ka, ga, fa, va, sa, za, ∫a, *Z*a, ma, na/ with a mean duration of 406.57 ± 106.61 ms (refer to Table 3 for details). Complete silent parts of the waveforms from both the onset and offset of each consonant syllable were identified by looking at time waveforms and spectrograms and then were manually removed. To verify whether this processing does not affect the perception of consonants, each processed consonant was presented 10 times in a random order in quiet to five adult NH listeners. The processed consonants were accepted as stimuli if they were identified with 99% accuracy. Then, consonants were processed by the AI-Gram to intensify the target frequency and time ranges with a 6 dB gain, as described below in the AI-Gram section.

**Table 3:** Target frequency and time ranges of consonants on which a + 6 dB gain was applied by the AI-Gram processing.

**Consonant**	**Duration of**	**Onset of vowel from the**	**Target frequency**	**Target time**
	**consonant [ms]**	**beginning of consonant [ms]**	**[kHz]**	**[ms]**
/pa/	240	59	0.3–7.4	10–40
/ba/	331	32	0.3–4.5	10–25
/ta/	338	96	3–7.4	50–70
/da/	240	43	4–7.8	15–25
/ka/	447	100	1.4–2	50–70
/ga/	348	52	1.4–2	30–50
/ma/	350	112	0.5–1.3	50–80
/na/	400	107	1.5–2.2	30–80
/fa/	548	180	0.6–2.2	45–70
/va/	349	88	0.6–1.4	20–50
/sa/	501	202	3.9–7.8	65–100
/za/	501	197	3.6–7.8	40–70
/∫a/	549	238	2–3.7	80–200
/*Z*a/	550	260	1.9–3.7	75–175

*Total durations of each consonant and the onset of the vowel sound /a/ from the beginning of each consonant were also given. The entire range of the frequency that was used for the identification of the target frequency range was 0 to 8 kHz. The target time ranges indicate the consonant temporal duration from the onset of the vowel /a/ in millisecond (ms)*.

For consonant recognition measures, the presentation level of each consonant was adjusted independently for each listening condition (left ear alone, right ear alone, and both ears) and was set to the “most comfortable” loudness level (MCL) in dB(A) SPL. The subjects were seated in the calibrated position in the sound field at 0° azimuth to the speaker, and MCL was determined using 5 dB increments according to the Cox loudness rating scale (Cox, 1995). The MCL was established utilizing the first ten unprocessed consonants from the stimuli listed above in quiet. The MCL for each listening condition is listed in Tables 1, 2.

Consonant recognition testing was performed with the speech level fixed at the MCL, and the speech-shaped noise was set to the level that yielded two signal-to-noise ratios or SNRs (+5 dB and +10 dB SNR). The SNRs were calculated with a linear rms level of speech input as a reference (i.e., 0 dB full-scale rms) after the AI-Gram signal processing on the target ranges. Speech level was individually scaled based on the subject's MCL. To achieve two SNRs, the noise level was adjusted, relative to the speech level. The speech-shaped noise was generated by combining long-term average spectrum of concatenated speech from 10 IEEE sentences to white noise (duration: 3 s and sampling frequency was 44,100 Hz using Praat). This noise masker was added to the unprocessed and AI-Gram-processed consonants to generate the designated SNRs. Speech-shaped noise was used because the information needed to identify individual phonemes occurs over a very short time frame, and it was reasoned that fluctuations present in maskers might lead to undue variability in performance. The choice of these SNRs was based on our preliminary studies with bimodal users and was used to validate the benefits of AI-Gram processing as well as avoid floor and ceiling effects (Yoon et al., 2019). The sum of speech signal and masking noise was filtered with a bandpass filter of 100–7,500 Hz before presentation to equalize the bandwidth. The overall presentation level of the bandpass filtered output (i.e., speech plus noise) was scaled to the subject's MCL. For each trial of speech tests conducted, the masker commenced 500 ms before the onset of the target speech and continued for 500 ms after the target offset, with cosine onset and offset ramps of 100 ms applied.

### AI-gram processing on the target frequency and time ranges

**Figure 2:**
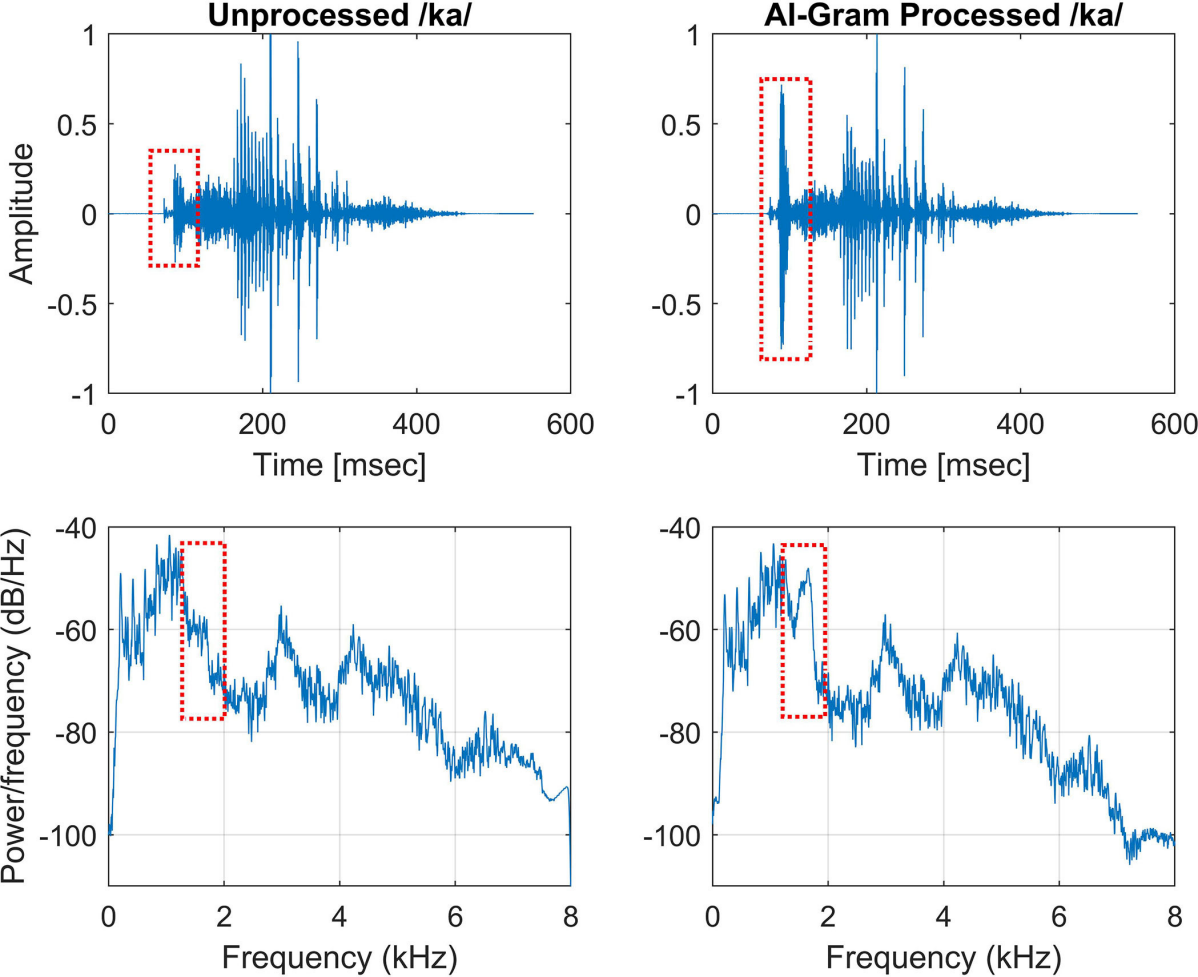
Timewave form (top panels) and power spectrum (bottom panels) before (left column) and after AI-Gram processing (right column) on the target frequency and time ranges for /ka/. Dotted squares indicate unprocessed and AI-Gram processed parts of the signal.

We used the same target frequency and time ranges that were used in our previous consonant perception study in NH listeners (Yoon, 2021). Figure 2 shows spectrograms before and after AI-Gram processing for /ka/. The squares indicate the target frequency and time ranges. The dotted vertical lines indicate the onset of the vowel /a/. The AI-Gram was originally developed by Li et al. (2010, 2012). We implemented the AI-Gram on the MATLAB platform for our conditions (MATLAB, 2013). Detailed procedures of the AI-Gram construction can be found in Yoon (2021). In brief, using a low-pass and high-pass filtering scheme (IIR second-order Butterworth with 12 dB/oct roll off and a zero-phase shift for both filters), the target frequency ranges were identified for each consonant by finding the frequency regions responsible for significant change in consonant recognition. For example, /ka/ was presented and perception scores significantly improved (from 40 to 90%) when the low-pass filter cutoff was moved from 1.4 to 1.5 kHz. So, the lower edge of the target frequency would be 1.4 kHz. When the high-pass filter cutoff was moved from 2.0 to 2.1 kHz, the recognition of /ka/ significantly dropped (from 90 to 40%). So, the upper edge of the target frequency would be 2.0 kHz. Therefore, the final target frequency range would be 1.4–2.0 kHz. Analogously, using a truncation approach, we identified the target time ranges for each consonant by finding the time segment of the consonant responsible for significant change in consonant recognition. The initial duration of each consonant was 3% of the total duration from the onset (i.e., the remaining 97% of the consonant was truncated out), so that minimal consonant information was presented. The duration of the consonant was increased by 1 ms when a participant's response was incorrect. If perception scores for */*ka*/* dropped significantly (i.e., 50%) when the time-truncation point increased from 50 to 70 ms from the onset of the vowel /a/, it suggested that important temporal cues resided within the 50- to 70-ms time window. Again, these target frequency and time ranges used for this study were obtained from NH listeners in the binaural hearing condition and in quiet (Yoon, 2021). After identifying the target frequency and time ranges for each of the 14 consonants using the AI-Gram, we applied a 6 dB gain to those target frequency and time ranges for each consonant (i.e., other frequency and time regions for each consonant were intact). The completed AI-Gram processing was then verified by five adult NH listeners. Verification procedure can also be found in Yoon (2021). Table 3 lists the resultant target frequency and time ranges to which we added a 6 dB gain for all 14 consonants. In Table 3, the target time range for /ba/ is 10–25 ms, indicating a temporal duration of /b/ from the onset (32 ms) of the vowel, relative to the beginning of the consonant syllables.

### Procedures

For both BCI and BHA groups, consonant recognition was measured in aided left ear only, aided right ear only, and both aided ears at a fixed +5 dB and +10 dB SNR and in quiet conditions. Subjects were seated in a single-walled, sound-treated booth (Industrial Acoustics Company) that was directly facing the loudspeaker (RadioEar SP90; frequency response: 125–8 kHz with a sensitivity of 94 dB/W; total harmonic distortion is <1% at 1 W and <5% at rated power) at 0° azimuth 1 m away. Subjects were tested with their CI or HA turned on. The device settings were programmed or mapped and were verified by their audiologist. An audiovisual inspection was performed on the device prior to testing. The hearing device on the non-tested ear was off and removed, and the non-tested ear was occluded with a foam hearing protective device (i.e., a single-sided earplug). Prior to testing, subjects were familiarized with the stimuli and the required task (15 min each for the unprocessed and AI-Gram processed consonants) was binaurally provided in quiet. During testing, subjects were instructed to identify the consonant presented by selecting the corresponding graphic symbol on a computer monitor. Subjects could repeat the stimulus up to three times. They were instructed to select the consonant they heard or make their best guess if they were unsure. Each consonant was presented 10 times at each SNR. The order of the consonants and SNR presented was randomized. This complete protocol was administered in two sessions: before AI-Gram (i.e., without signal enhancement) and after AI-Gram (i.e., with signal enhancement). To avoid the sequence effect, half of the subjects in each group were tested first without the signal enhancement, whereas the other half were tested first with the signal enhancement. In addition, the order of listening condition (i.e., left ear, right ear, and both) was randomized within the group: three subjects were first tested with left ear, three were first tested with right ear, and remaining subjects were first tested with both ears together. The complete testing protocol including familiarization and breaks approximately took 9 h per subject, requiring three separate visits.

### Data analysis

**Figure 3:**
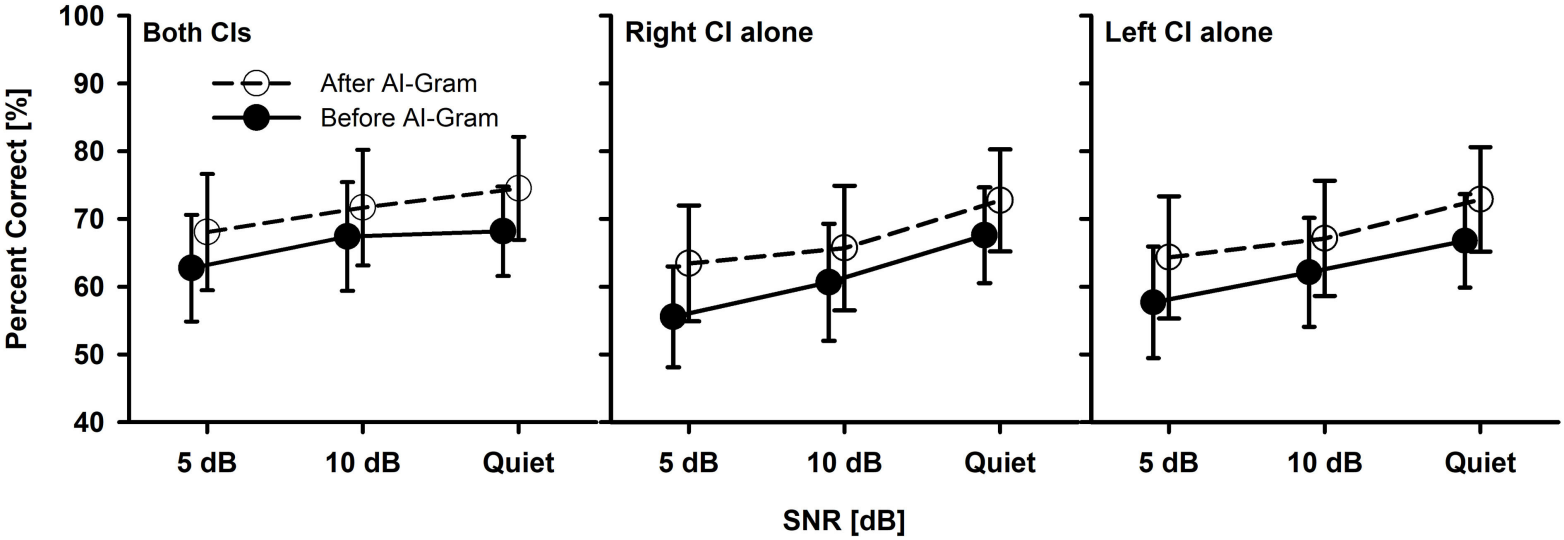
Mean percent correct scores with standard error of the BCI group between before and after the AI-Gram processing for both CIs, right CI alone, and left CI alone as a function of SNR. For a better visualization of the error bars, datapoints for the before AI-Gram condition were slightly shifted to the left.

**Figure 4:**
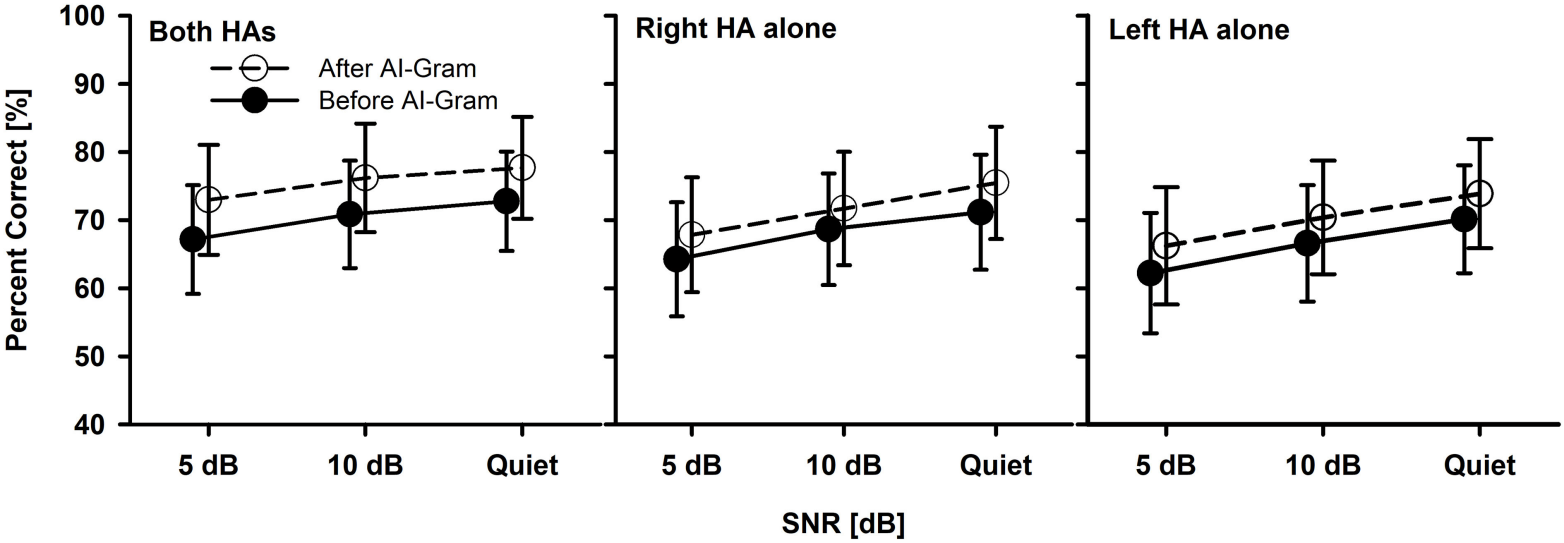
Mean percent correct scores with standard error of the BHA group between before and after the AI-Gram processing for both HAs, right HA alone, and left HA alone as a function of SNR. For a better visualization of the error bars, datapoints for the before AI-Gram condition were slightly shifted to the left.

**Figure 5:**
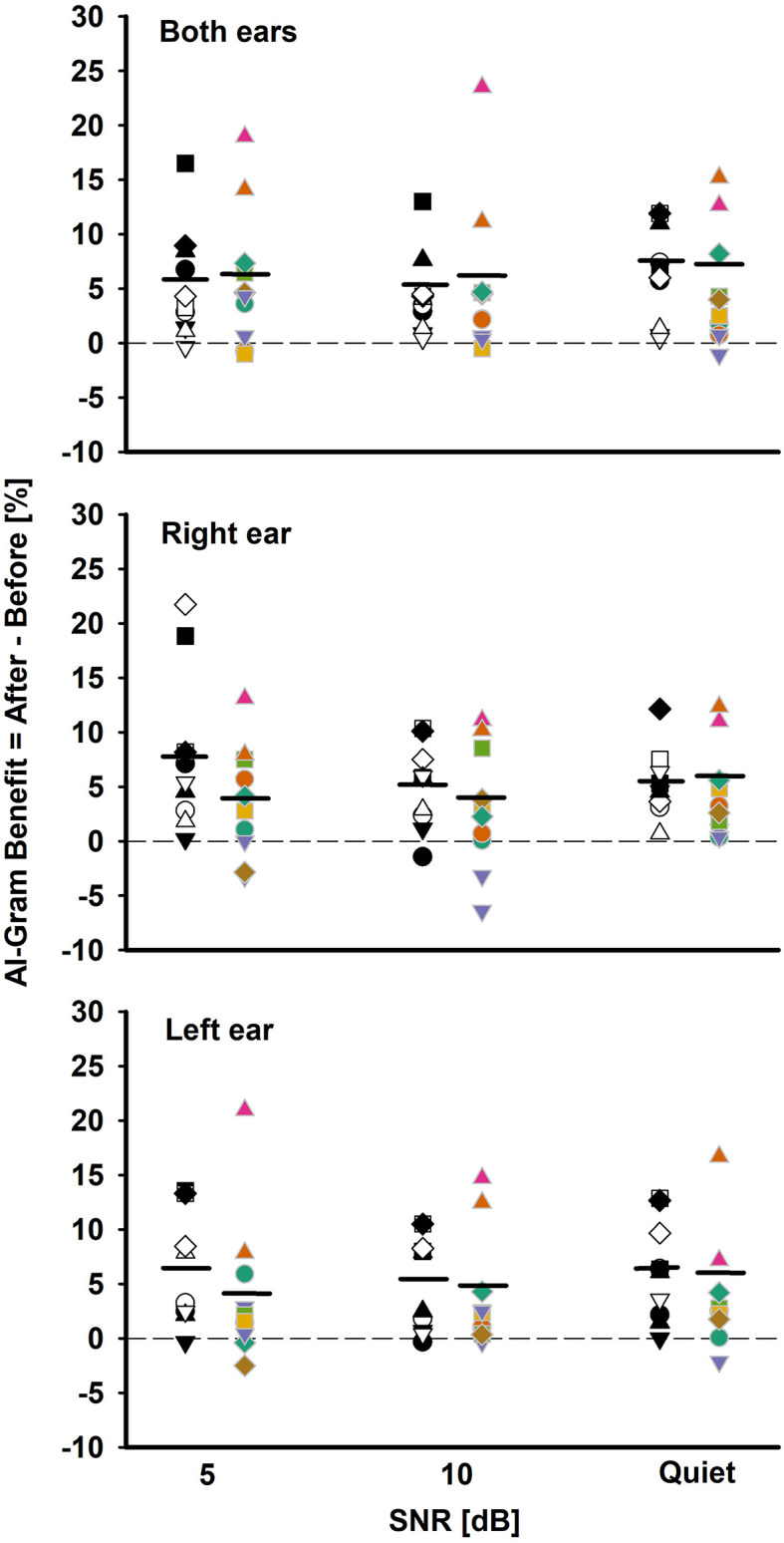
Individual and mean AI-Gram benefit for the BCI group (white and black symbols in the left column) and BHA group (colored symbols in right column) for each SNR in both ears, right ear alone, and the left ear alone listening conditions. Mean AI-Gram benefits are denoted by solid horizontal lines. Zero reference is indicated by dotted lines.

**Figure 6:**
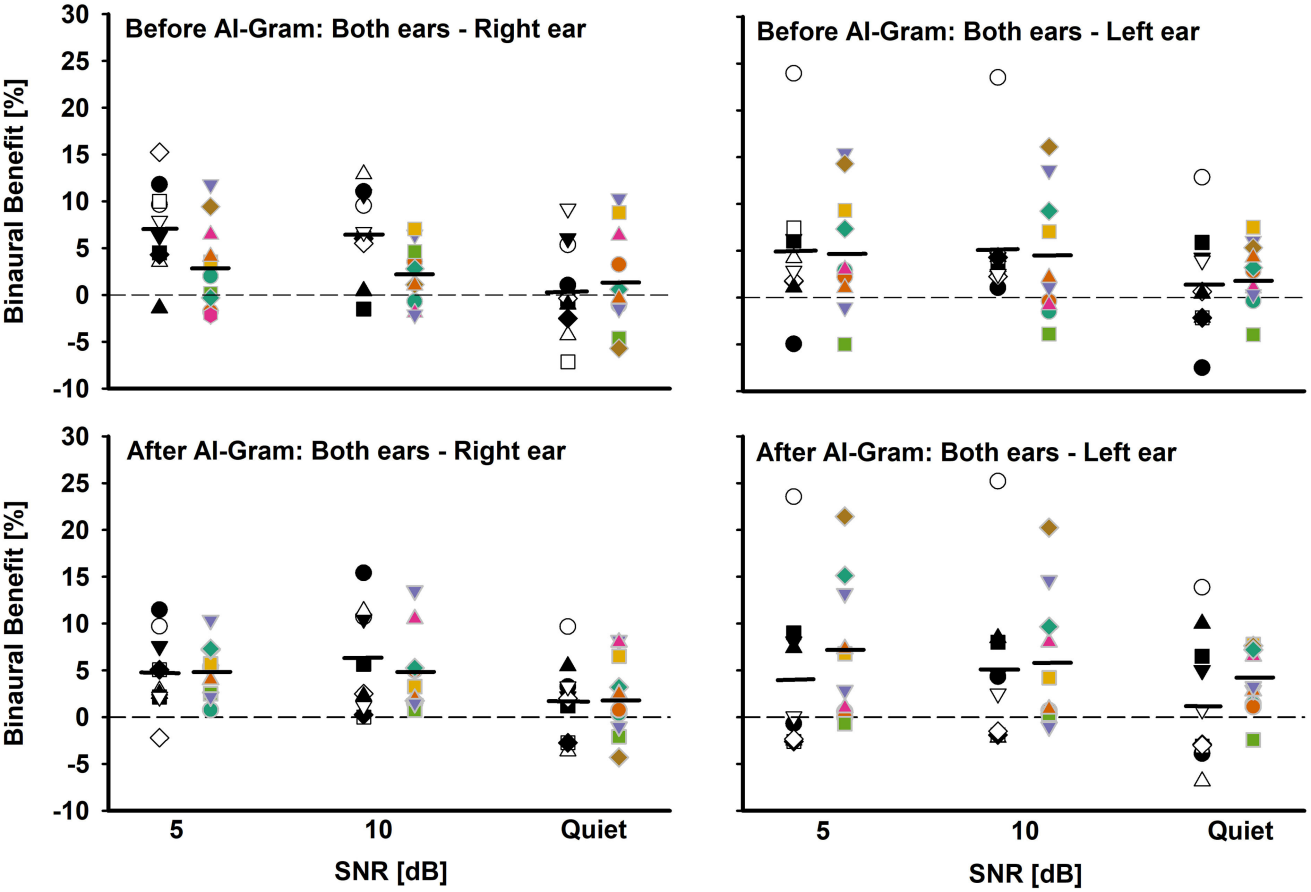
Individual and mean binaural benefit before (top panels) and after (bottom panels) AI-Gram processing for each SNR. On each panel, the BCI group is denoted by white and black symbols in the left column and BHA group is denoted by colored symbols in right column. Two left panels present binaural benefit against right ear and two right panels present binaural benefit against left ear. Mean binaural benefits are denoted by solid horizontal lines. Zero reference is indicated by dotted lines.

Before statistical analyses, we checked the floor and ceiling effects to determine the three major outcome measures accurately (i.e., the AI-Gram benefit, binaural benefit, and binaural interference). For example, if the participant got a score of 100% at +5 dB SNR, we would not expect any change in performance at +10 dB SNR. Similarly, if they had a score of 100% in a monaural listening condition, we would not expect an improvement in a binaural listening condition. To determine the mean difference before and after the AI-Gram processing for both BCI (Figure 3) and BHA (Figure 4) groups, a three-way repeated measures analysis of variance (ANOVA) was performed to determine the main effects of the three within-subject factors: the AI-Gram, listening condition (both ears, right ear alone, and left ear alone), and SNR. To determine the mean difference between BCI and BHA groups in the AI-Gram benefit (Figure 5) and binaural benefit (Figure 6), we performed a three-way mixed ANOVA with one between-subject factor (i.e., group) and two within-subject factors (i.e., the AI-Gram and SNR). The results of all statistical analyses were assessed against an alpha level of 0.05 with a two-tailed test. Planned multiple comparisons were performed using an overall alpha level of 0.05 with the Bonferroni correction.

## Results

### Significant AI-gram benefit in BCI group

Figure 3 shows the mean consonant recognition score for the BCI group before and after the AI-Gram processing as a function of SNR and listening condition (both CIs, right CI alone, and left CI alone). Normality (Shapiro–Wilk) test and equal variance (Brown–Forsythe) test were all passed. *Post-hoc* powers for the statistical analyses ranged from 0.93 to 0.99 for both AI-Gram processing and SNR, which were calculated with mean percent scores and standard deviations, alpha of 0.05, and a sample size of 10. Consonant recognition significantly improved after the AI-Gram processing compared to scores before the AI-Gram processing, *F*_(1, 36)_ = 27.78, *p* = 0.001. Effect sizes for the AI-Gram benefit were 0.40, 0.34, and 0.38 for both CIs, right CI alone, and left CI alone, respectively. Based on the effect size guidelines in the field of hearing science (Gaeta and Brydges, 2020), these effect sizes are small. The performance was significantly affected by the listening condition, *F*_(2, 36)_ = 3.76, *p* = 0.04, and different SNRs, *F*_(2, 36)_ = 15.47, *p* = 0.001. The interaction was significant only between the listening condition and SNR, *F*_(4, 36)_ = 4.61, *p* = 0.004. Pairwise multiple comparisons with the Bonferroni correction were performed. The results showed that any pair between before and after AI-Gram at each SNR was significantly different for each listening condition (*p* < 0.01). Multiple comparisons also showed that scores with both CIs were significantly different from those with the right CI alone (*p* = 0.003) but not with the left CI alone (*p* = 0.36). Differences between the right CI and left CI were not significant (*p* = 1.0). Pairwise multiple comparisons showed that scores at 5 dB SNR were significantly different from those at 10 dB SNR (*p* = 0.005) and at quiet (*p* = 0.005). Differences between 10 dB SNR and quiet conditions were marginally significant (*p* = 0.04).

### Significant AI-gram benefit in BHA group

The mean consonant recognition score of the BHA group is presented in Figure 4. The normality test was failed for the binaural listening condition, but both the normality and equal variance tests were passed for all other conditions. *Post-hoc* powers for the statistical analysis ranged from 0.50 to 0.64 for the effect of the AI-Gram processing. For the SNR effect, *post-hoc* powers ranged from 0.67 to 0.99. These powers were calculated with mean percent scores and standard deviations, alpha of 0.05, and a sample size of 10. Compared to the before AI-Gram processing, consonant recognition scores significantly improved after the AI-Gram processing, *F*_(1, 36)_ = 7.17, *p* = 0.03. The effect sizes for the AI-Gram benefit were 0.29, 0.23, and 0.16 for both HAs, right HA alone, and left HA alone, respectively. According to Gaeta and Brydges's guidelines for effect size, these are considered small effect sizes. The consonant recognition also was significantly affected by the listening condition, *F*_(2, 36)_ = 5.20, *p* = 0.02 and SNRs, *F*_(2, 36)_ = 9.70, *p* = 0.001. However, all interactions were not significant (*p* > 0.05). Pairwise multiple comparisons with the Bonferroni correction showed that any pair between before and after AI-Gram at each SNR was significantly different for each listening condition (*p* < 0.01). Multiple comparisons also showed that scores with both HAs were significantly different from those with the right HA alone (*p* = 0.01) but not with the left HA alone (*p* = 0.22). Differences between the right HA and left HA were not significant (*p* = 1.0). Pairwise comparisons also showed that scores at 5 dB SNR were significantly different from those at 10 dB SNR (*p* = 0.02) and in quiet (*p* = 0.03). However, differences between 10 dB SNR and quiet conditions were not significant (*p* = 0.10).

### Comparisons in AI-gram benefit between BCI and BHA groups

One of our interests was to compare the ability of the BCI and BHA groups for utilizing the intensified target frequency and time ranges for consonant recognition. This comparison can provide an insight regarding the ability of spectrotemporal integration between the groups. We computed the AI-Gram benefit by subtracting the listener's performance score after the AI-Gram processing from the listener's performance score before processing. Figure 5 depicts individual AI-Gram benefits for the BCI (black and white symbols in the left column) and BHA (colored symbols in the right column) listeners with the group mean, indicated by solid horizontal lines as a function of SNR and the listening condition.

With the mean data, we performed a three-way mixed ANOVA with one between-subject factor (i.e., the group) and two within-subject factors (i.e., listening condition and SNR). Normality and equal variance tests were all passed. The AI-Gram benefit was not significantly different between the groups, *F*_(1, 180)_ = 3.49, *p* = 0.06 and across the listening conditions, *F*_(2, 180)_ =0.16, *p* = 0.85. The main effect of the SNR was not significant either, *F*_(2, 180)_ = 0.70, *p* = 0.50. No interactions among the three factors were significant (*p* > 0.05).

As for the individual data, large variability exists regardless of the group, the listening condition, and SNR. For both ears, the BCI listeners received the AI-Gram benefit from −0.8 to 17% (average 5.13%) over SNR, whereas the BHA listeners received the benefit from −1 to 23% (average 5.2%). In total, seven BCI listeners received more than a 5% benefit, aggregated over SNRs, compared to four BHA listeners. One BCI listener experienced negative effects (-0.8%) of the AI-Gram processing at 5 dB SNR, whereas three BHA listeners had negative values (around −1%) for each of the SNRs. It should be noted that AI-Gram benefits from BHA4 and BHA9 participants, indicated by filled upward pointing triangles, were relatively greater than others.

For the right ear alone, the AI-Gram benefit for the BCI group ranged from −1 to 22% (average 5.9%) over the SNRs, whereas the BHA listeners received the benefit from −6.6 to 13% (average 3.6%). In total, seven BCI listeners experienced more than a 5% benefit, aggregated over the SNRs, and all except one subject received a > 5% benefit with the right CI alone in both CI listening conditions, respectively. A total of seven BHA listeners received more than a 5% benefit. In total, four of them experienced more than a 5% benefit in the right HA alone as well as the listening condition using both HAs. One BCI group listener had a negative value (−1%) from the AI-Gram at 10 dB SNR. In total, four BHA listeners had negative values (−3.2 to −6.6% and average −4.1%) over the SNRs, and two of them experienced a negative effect from the AI-Gram processing in both HA listening conditions.

For the left ear alone, the BCI listeners received the AI-Gram benefit from −0.5 to 13.5% (average 5.9%) over the SNRs, whereas the BHA listeners received the benefit from −2.5 to 21% (average 3.83%). In total, six BCI listeners received a greater than 5% benefit and five of them experienced this benefit in the right CI alone as well as the listening condition using both CIs. A total of three BHA listeners had more than a 5% benefit and two of them experienced the benefit in both the left HA alone and both HA listening conditions. A total of two BCI listeners had a negative effect (−1%) from the AI-Gram at 5 dB and 10 dB SNR, and neither of them experienced a negative effect in both CI listening conditions. A total of four BHA listeners experienced negative values (−0.2 to −2.9% and average −1.45%) over the three SNRs, and one of them also experienced a negative AI-Gram benefit in both HA listening conditions.

### Binaural benefit between BCI and BHA groups

Another aim of this study was to assess binaural benefit (indicated by a difference in percent correct consonant scores between binaural hearing and each monaural ear) from AI-Gram processing on the target frequency and time ranges in consonant recognition. Figure 6 shows individual binaural benefit before (top panels) and after (bottom panels) the AI-Gram processing. The two left panels present binaural benefit for the right ear, whereas the two right panels present binaural benefit for the left ear. On each panel, the BCI listeners are indicated by black and white symbols in the left column, and BHA listeners are indicated by colored symbols in the right column. The group mean is also denoted by solid horizontal lines.

With the mean data, we performed a three-way mixed ANOVA with one between-subject factor (i.e., the group) and two within-subject factors (i.e., the AI-Gram and SNR). Normality and equal variance tests were all passed. The binaural benefit was not significantly different between the groups, *F*_(1, 59)_ = 0.52, *p* = 0.87 and between the before and after AI-Gram processing, *F*_(1, 59)_ = 1.34, *p* = 0.25. The main effect of the SNR was significant, *F*_(2, 59)_ = 3.35, *p* = 0.04. All interactions among the three factors were not significant (*p* > 0.05).

The individual data before the AI-Gram processing for the right ear (top-left panel) show that the BCI listeners received binaural benefit from −7.6 to 15.9% (average 4.8%) over the SNRs, whereas the BHA listeners experienced binaural benefit from −5.8 to 11.8% (average 2.2%). In total, five BCI group listeners and three BHA listeners received more than a 5% binaural benefit, which were aggregated over the SNRs. However, seven BCI listeners (subjects 1, 4, 5, 6, 7, 8, and 9) experienced binaural interference with a magnitude of −0.5 to −7.6% (average −3.7%). In total, four BHA listeners also experienced binaural interference, with a magnitude of −0.5 to −1.8% (average −1.3%). The binaural benefit for the left ear (top-right panel) shows that the BCI listeners received binaural benefit from −7.5 to 24.0% (average 3.9%) over the SNRs, whereas the BHA listeners experienced binaural benefit from −4.0 to 16.1% (average 4.0%). One BCI group listeners and four BHA listeners received more than a 5% binaural benefit, which were aggregated over the SNRs. However, one listener in each group experienced binaural interference with a magnitude of −3.8 and −4.3%, respectively.

After the AI-Gram processing, binaural benefit for BCI listeners for the right ear (bottom-left panel) ranged from −3.7 to 15.4% (average 4.2%) over the SNRs, whereas the BHA listeners experienced binaural benefit from −4.3 to 10.4% (average 3.9%). In total, three BCI group listeners and four BHA listeners received more than a 5% binaural benefit, which were aggregated over the SNRs. None of listeners in either group experienced binaural interference. The binaural benefit for the left ear (bottom-right panel) shows that the BCI listeners received binaural benefit from −6.9 to 25.2% (average 3.3%) over the SNRs, whereas the BHA listeners experienced binaural benefit from −2.5 to 21.5% (average 5.5%). In total, four BCI group listeners and five BHA listeners received more than a 5% binaural benefit, which were aggregated over the SNRs. However, five BCI listener experienced binaural interference with a magnitude of −0.1 to −3.9 and one BHA listener experienced a binaural interference of −1%.

### Correlation between binaural benefit and audiological/demographic data

To determine whether the binaural benefit was associated with audiological and demographic factors, we performed a Pearson's correlation analysis based on the Bonferroni correction for six multiple correlations with a significant level of 0.0083 (i.e., 0.05/6). The binaural benefit was not significantly correlated with any factors such as years of BCI experience, age, age of implantation, monaural performance alone, the performance difference between ears, and scores averaged between ears (*p* > 0.05). For the BHA group, a pure-tone average over 0.25, 0.5, and 0.75 kHz was significantly correlated with binaural benefit for before (*r* = 0.54, *p* = 0.002) and after (*r* = 0.60, *p* < 0.001) AI-Gram processing. However, the binaural benefit was not significantly correlated with any other factors such as age, monaural performance alone, the performance difference between ears, and scores averaged between ears (*p* > 0.05).

## Discussion

This study was designed to answer the following three questions for both groups and then compare the BCI to the BHA findings (1) Was there a significant AI-Gram benefit at varying SNRs? (2) Was there a binaural benefit improvement with AI-Gram processing? (3) Was binaural interference reduced with AI-Gram processing? Our study showed a small (~ 5%) but statistically significant improvement in consonant recognition after AI-Gram processing. The results demonstrated that listening condition (i.e., monaural or binaural) and SNR had a significant impact on consonant recognition for both groups. However, group differences in the mean AI-Gram benefit were not significant. Binaural benefit was found for both groups but was not significantly different between the groups, nor was binaural benefit significantly improved after AI-Gram processing for either group. These results suggest that the target frequency and time ranges that were enhanced by the AI-Gram contribute to consonant recognition improvement, regardless of the listening condition or listening technology (i.e., BCI or BHA). However, the enhanced frequency and time ranges contributed less additional improvement to binaural benefit for both groups.

### Target frequency and time ranges and consonant enhancement

In this study, we determined whether there was a significant AI-Gram benefit at varying SNRs, in different listening conditions for each group and then compared the BCI findings to the BHA findings. The results revealed that consonant recognition was significantly improved after the AI-Gram processing for both the BCI and BHA groups. However, differences in the mean AI-Gram benefit between groups were not significant. Our results are comparable to those reported by Yoon et al. (2019) and Yoon (2021). Yoon et al. (2019) measured the AI-Gram benefit in consonants with bimodal CI users. The mean AI-Gram benefits were 8.7, 4.3, and 7.7% in the bimodal, CI alone, and HA alone listening conditions, respectively, averaged over the same three SNRs used in this study. Yoon (2021) also measured the consonant AI-Gram benefit with NH listeners at −30, −20, and −10 dB SNR under a binaural listening condition. The mean AI-Gram benefit was 7.8%, averaged over the SNRs. Due to the different range of SNR tested, direct comparisons between the current and Yoon (2021) studies should not be made even though the same AI-Gram processing was employed for the same sets of consonant recognition.

As for the individual data (Figure 5), a large variability in the AI-Gram benefit exists regardless of the monaural or binaural listening condition for both groups. The AI-Gram benefit ranged from −1.0 to 22% for the BCI group and from −6.6 to 23% for the BHA group. These data indicate that some listeners have a better ability to detect and process spectral and temporal cues of AI-Gram processed consonants than others. This difference in detection ability could stem from many factors discussed in the Introduction such as insertion depth of the electrode array for the CI users, length of acoustic and electric stimulation, duration of hearing loss, length of auditory deprivation, and the vitality of the ascending and descending auditory pathways.

As mentioned above in the Result section, two BHA group participants (BHA4 and BHA9) received relatively greater AI-Gram benefit across listening conditions and SNRs. We initially thought that better residual hearing at lower frequencies could be a potential contributing factor. However, BHA4 has very poor residual thresholds (65, 90, and 90 dB HL) at 0.5, 0.75, and 1 kHz. It is unclear what caused the two participants to be outliers. More systematic investigation is warranted to determine which factors limit or enhance the ability to detect spectral and temporal cues. These individual datasets may lead to developing individualized treatment options for clinical populations.

There are two interesting observations to note. First, there is a trend that listeners in the BCI group who received the AI-Gram benefit in the monaural CI listening condition also experienced a similar magnitude of the AI-Gram benefit in the binaural CI listening condition. In total, nine out of 10 BCI listeners fall in this category. The BHA group shows a similar trend, but the trend is not as obvious as the BCI group. In total, six BHA listeners fall in this category. Our results showed that the AI-Gram benefit followed the better hearing ear alone which is suggestive of the contribution of an ear dominance mechanism. An ear dominance mechanism results in information presented to one ear being perceived while information presented to the opposite ear is suppressed. Reiss et al. found that the ear dominance mechanism is common in BHA users who have a narrower fusion range across HA ears, which are assessed with pure tones (Reiss et al., 2016).

Second, there is a negative effect from the AI-Gram processing seen in both groups. Three BCI listeners experienced negative effects (<20%) from AI-Gram processing, which were aggregated over listening conditions and the SNRs, whereas six BHA listeners experienced effects from AI-Gram processing. It seems that the negative effect of the AI-Gram processing is independent of the listening condition for both groups. Listeners who experienced negative values in the monaural listening condition did not experience negative values in the binaural listening condition. Similarly, listeners who experienced negative values in the binaural listening condition did not necessarily experience the negative effect in their monaural listening condition. One technical concern can explain this mild negative effect from the AI-Gram. Even though the AI-Gram processed consonants were verified by five NH listeners (Yoon, 2021), there is a possibility that the target frequency and time ranges enhanced by +6 dB via the AI-Gram may create sound distortions for the BCI and BHA group listeners. Li et al. reported multiple distortion cases in NH listeners when the target frequency and time ranges were intensified by a value higher than +6 dB (Li et al., 2010, 2012). Since these negative effects of the AI-Gram processing were not large, the findings might be due to random errors, rather than systematic interferences.

### Binaural integration

In this study, we determined whether there was an improvement to binaural benefit with AI-Gram processing for each group and then compared the BCI findings to the BHA findings. The results revealed that while there was a significant binaural benefit, there was not an improvement in binaural benefit with the addition of AI-Gram processing. It is interesting to note that results revealed a trend in which listeners in each group who demonstrated AI-Gram benefit in the monaural listening condition also experienced a similar magnitude of AI-Gram benefit in the binaural listening condition. As discussed in the previous section, this trend could be explained by an ear dominance mechanism.

Our finding of no significant additional binaural benefit with AI-Gram processing is consistent with the report by Yoon et al. who tested NH listeners for consonant recognition using the same AI-Gram, which is used in this study (Yoon et al., 2019). They reported a 1.8 and 1.6% binaural benefit before and after the AI-Gram processing, respectively, over SNRs (−30, −25, −20, −15, and −10 dB). The results from both studies simply mean that a similar AI-Gram benefit occurred regardless of listening with one or two ears. Also, though the mean binaural benefits in this study were <5%, five BCI listeners and four BHA listeners received more than a 5% binaural benefit before the AI-Gram processing whereas four BCI listeners and five BHA listeners experienced more than 5% benefit after the AI-Gram processing. Reiss et al. demonstrated this highly individualized ability to integrate frequency information across ears by measuring fusion ranges and spectral averaging in different listening groups. They reported very large variability in binaural spectral fusion (as much as 3–4 octaves, compared to 0.1 octaves in NH listeners) in BCI (Oh and Reiss, 2020) and BHA users (Reiss et al., 2016; Oh and Reiss, 2017). Our individual data, along with data from Reiss's studies, suggest that hearing technology (i.e., BCI or BHA) is not a requirement to optimize the integration process. The individual variability in additional binaural benefit with AI-Gram processing for both groups shown in our study also suggests that integration of consonant information is highly dynamic and listener specific. This variability in additional binaural benefit with AI-Gram processing in quiet and in noise seems to be related to the ability of listeners in both groups to integrate spectrotemporal cues. Further research is needed to better understand the correlation between the integration ability and variability of binaural benefit in speech perception for those with two hearing devices.

### Binaural interference

In the study, we determined whether binaural interference was reduced with AI-Gram processing for each group and then compared the BCI findings to the BHA findings. In addition, three BCI listeners and six BHA listeners showed negative effects from AI-Gram processing, which was independent of the listening condition for both groups. The findings showed that seven BCI listeners experienced binaural interference before AI-Gram processing and five listeners after AI-Gram processing. A total of six BHA listeners experienced binaural interference before AI-Gram processing and three listeners after AI-Gram processing. For both groups, the number of listeners who experienced binaural interference was reduced after the AI-Gram processing, but the range of the interference remained relatively constant. The number of binaural interference cases was reduced more in BHA listener group than in the BCI listener group.

The binaural interference of <5% may be due to random errors, but we have subjects with more than 5% interference from both groups. The underlying factors for binaural interference are unclear. One potential factor in the BCI group would be either pre-operative asymmetric hearing loss or the duration of hearing loss. Goupell et al. showed that all nine of their BCI adult users with either an early onset of deafness or an asymmetric hearing loss, which could have resulted in significant periods of auditory deprivation, experienced significant interference in speech perception in noise and quiet (Goupell et al., 2018). With similar testing conditions, other research showed that all four of their high-performing, experienced, late-deafened BCI users received significant binaural benefit without any binaural interference (Bernstein et al., 2019). Binaural interference can be considered contralateral masking. That is, speech acoustics presented to one ear negatively affects the ability of the opposite ear to detect and integrate acoustic speech cues. Aronoff et al. have shown a negative correlation between contralateral masking and speech perception in BCI users (Aronoff et al., 2015). A more systematic investigation on the relationship between contralateral masking and binaural interference is warranted. As for the BHA listeners, Reiss et al. showed that BHA listeners with a broad fusion range (or poorer ability to discriminate tone difference across ears) experienced binaural interference in vowel perception (Reiss et al., 2016; Oh and Reiss, 2017).

Listeners who experienced negative values in the monaural listening condition did not experience negative values in the binaural listening condition. Similarly, listeners who experienced negative values in the binaural listening condition did not necessarily experience the negative effect in their monaural listening condition.

### Binaural benefit, demographic, and audiological factors

Our correlation analyses showed that binaural benefit in the BCI group was not significantly correlated with any other factors such as years of BCI experience, subject age, age of implantation, monaural performance alone, the performance difference between ears, and scores averaged between ears. However, numerous studies have reported that the age of implantation affected the success rate of binaural hearing capabilities across the lifespan due to the complex nature of audiological development (Grieco-Calub and Litovsky, 2010; Van Deun et al., 2010; Litovsky and Gordon, 2016). We cannot relate our correlation analyses with these findings because we did not exclude subjects based on the length of auditory deprivation between hearing loss onset and intervention. This fact may limit a correlation between binaural benefit and the age of implantation. For the BHA group, a pure-tone average over 0.25, 0.5, and 0.75 kHz was significantly correlated with binaural benefit both before and after AI-Gram processing. However, the binaural benefit was not significantly correlated with any other factors such as age, monaural performance alone, the performance difference between ears, and scores averaged between ears. Age is also a significant factor for HA satisfaction, which was assessed with the Hearing Aid Satisfaction Questionnaire (Korkmaz et al., 2016). Our correlation analyses were performed with a limited sample size in both groups, which limits us in determining the correlations of the binaural benefit in speech perception with other important patient and audiologic related factors.

### Limitations and future plan

This study has several limitations. First, both BCI and BHA subjects may need subject-specific target frequency and time ranges rather than generic ones, which we obtained from NH subjects. As BCI users have the different degree of within and across spectral mismatch between place frequency and programmed frequency for each electrode (Kan et al., 2013; Canfarotta et al., 2020; Bernstein et al., 2021), the spectral mismatches affect the identification of the target frequency and time ranges. For BHA users, there are substantial differences in the degree of residual hearing within and across patients (Sheffield and Zeng, 2012; Visram et al., 2012). The target frequency and time range can significantly be affected by these audiometric differences. To capture these differences across patients, the identification of the target frequency and time ranges is needed on an individual basis. Second, the small sample size of each group limits the generalization of the current results. Studies have shown that increased length of time using CIs or HAs leads to better scores on various audiometric tests (Litovsky et al., 2006; Grieco-Calub and Litovsky, 2010). Increased sample size would allow for the grouping of subjects by the length of time they used the device, which would generate data for comparison and allow for more solid conclusions. Larger sample size also allows us to match demographic and audiometric factors between groups that result in better baseline equivalence. *Post-hoc* power analyses with the smallest effect size of the CI group (η^2^ = 0.34) showed a desired sample size of 18 for a power of 0.9. The same analyses with the smallest effect size of the HA group (η^2^ = 0.16) required 24 subjects. Third, recordings of one female talker were used to limit the effect of different talkers on the identification of the target ranges. Using a single talker significantly underestimates the amount of talker variability that would be present in real listening situations. The target ranges might be very different, depending on different talkers. Finally, we used the single phonetic environment (consonant+/a/ vowel). Frequency-time regions that support robust perception of a consonant will be changed if different vowels with different positions of consonants (initial, medial, or final) are used as stimuli (Hayden, 1950; Harris, 1958). Currently, our laboratory has conducted a series of studies to determine the effect of frequency-to-place spectral mismatch in a CI ear, the effect of fitted center frequency mismatch between a HA ear and a CI ear, and the effect of different degrees of residual hearing in a HA ear on bimodal frequency importance function. In addition, a spectral integration and interference study is ongoing for vowel and consonant recognition with a manipulation of first and second formant frequencies. Our preliminary data show that binaural integration is better facilitated when fitted center frequencies between a HA ear and a CI ear are less mismatched, thus resulting in greater AI-Gram effect on the binaural benefit. Our long-term goal of the AI-Gram-based speech recognition studies was to develop algorithms for deep machine learning. For individuals with hearing loss or hearing devices, an individually tailored signal processing scheme is critical to optimize the performance of hearing devices. Our subject-by-subject and sound-by-sound identification scheme of the target and conflicting ranges for phonemes will generate necessary data to train the algorithms for deep machine learning. Another practical implication of improved consonant recognition, which is critical for lexical access (Toro et al., 2008), is a reduction in required listening effort and less expenditure of cognitive resources for speech perception (Peelle, 2018).

## Data availability statement

The raw data supporting the conclusions of this article will be made available by the authors, without undue reservation.

## Ethics statement

This study was reviewed and approved by Texas Tech University Health Sciences Center. Written informed consent was obtained from all participants for their participation in this study in accordance with the Declaration of Helsinki.

## Author contributions

Y-SY conceived and designed the study, conducted the experiments, analyzed the data, and wrote the draft of the manuscript. CD completed the manuscript by adding clinical literature review and components. Both authors contributed to the article and approved the submitted version.

## Funding

This work was partially supported by the American Hearing Research Foundation and the National Institutes of Health under R15DC019240 to Y-SY. The funders were not involved in the study design, collection, analysis, interpretation of data, the writing of this article, or the decision to submit it for publication.

## Conflict of interest

The authors declare that the research was conducted in the absence of any commercial or financial relationships that could be construed as a potential conflict of interest.

## Publisher's note

All claims expressed in this article are solely those of the authors and do not necessarily represent those of their affiliated organizations, or those of the publisher, the editors and the reviewers. Any product that may be evaluated in this article, or claim that may be made by its manufacturer, is not guaranteed or endorsed by the publisher.
